# Persistence survey of Toxic Shock Syndrome toxin-1 producing *Staphylococcus aureus *and serum antibodies to this superantigen in five groups of menstruating women

**DOI:** 10.1186/1471-2334-10-249

**Published:** 2010-08-23

**Authors:** Jeffrey Parsonnet, Melanie A Hansmann, Jon L Seymour, Mary L Delaney, Andrea M DuBois, Paul A Modern, Michaelle B Jones, John E Wild, Andrew B Onderdonk

**Affiliations:** 1 Dartmouth-Hitchcock Medical Center, One Medical Center Drive, Lebanon, New Hampshire, USA; 2 Channing Laboratory, Brigham & Women's Hospital, Harvard Medical School, 181 Longwood Ave, Boston, Massachusetts, USA; 3 The Procter & Gamble Company, 6010 Center Hill Ave., Cincinnati, Ohio, USA; 4 Hill Top Research Inc., Main & Mill St., Miamiville, Ohio, USA

## Abstract

**Background:**

Menstrual Toxic Shock Syndrome (mTSS) is thought to be associated with the vaginal colonization with specific strains of *Staphylococcus aureus *TSST-1 in women who lack sufficient antibody titers to this toxin. There are no published studies that examine the seroconversion in women with various colonization patterns of this organism. Thus, the aim of this study was to evaluate the persistence of *Staphylococcus aureus *colonization at three body sites (vagina, nares, and anus) and serum antibody to toxic shock syndrome toxin-producing *Staphylococcus aureus *among a small group of healthy, menstruating women evaluated previously in a larger study.

**Methods:**

One year after the completion of that study, 311 subjects were recalled into 5 groups. Four samples were obtained from each participant at several visits over an additional 6-11 month period: 1) an anterior nares swab; 2) an anal swab; 3) a vagina swab; and 4) a blood sample. Gram stain, a catalase test, and a rapid *S. aureus*-specific latex agglutination test were performed to phenotypically identify *S. aureus *from sample swabs. A competitive ELISA was used to quantify TSST-1 production. Human TSST-1 IgG antibodies were determined from the blood samples using a sandwich ELISA method.

**Results:**

We found only 41% of toxigenic *S. aureus *and 35.5% of non-toxigenic nasal carriage could be classified as persistent. None of the toxigenic *S. aureus *vaginal or anal carriage could be classified as persistent. Despite the low persistence of *S. aureus *colonization, subjects colonized with a toxigenic strain were found to display distributions of antibody titers skewed toward higher titers than other subjects. Seven percent (5/75) of subjects became seropositive during recall, but none experienced toxic shock syndrome-like symptoms.

**Conclusions:**

Nasal carriage of *S. aureus *appears to be persistent and the best predicator of subsequent colonization, whereas vaginal and anal carriage appear to be more transient. From these findings, it appears that antibody titers in women found to be colonized with toxigenic *S. aureus *remained skewed toward higher titers whether or not the colonies were found to be persistent or transient in nature. This suggests that colonization at some point in time is sufficient to elevate antibody titer levels and those levels appear to be persistent. Results also indicate that women can become seropositive without experiencing signs or symptoms of toxic shock syndrome.

## Background

Toxic shock syndrome (TSS) is a systemic disease of acute onset characterized by fever, hypotension, myalgia, rash, multiple-organ failure, and late desquamation of hands and feet [1]. It is associated with colonization with toxic shock syndrome toxin-1 (TSST-1)-producing *S. aureus *in the vagina during menstruation, or at other sites due to complications of a staphylococcal infection (especially skin or respiratory tract), or as a complication of a surgical procedure or other medical condition [2,3]. TSST-1, the most common such toxin, causes the vast majority (95%) of cases associated with menstruation and 40-60% of the nonmenstrual cases [4,5].

Menstrual Toxic Shock Syndrome (mTSS) has been associated with menstruation and tampon use. Despite the very low incidence of mTSS, the disease remains of interest, because tampons are widely used. Czerwicnski [6] reported in a recent descriptive research study that approximately 80% of the study participants (women under of the age of 41 from California) used tampons at some point during menstruation. It has also been reported recently that about 70% of women in the United States of America (USA), Canada and much of Western Europe use tampons at some point during menstruation [7].

Menstrual TSS is generally thought to be caused by *S. aureus *TSST-1 in a susceptible host [8,9]. TSST-1 is considered a superantigen (SAg), a class of very potent immune stimulators that interact with the immune system in a way that is different from conventional antigens. As a result, the magnitude of immune stimulation by a SAg is usually 10-500,000 fold higher than with convention antigens. This exaggerated release of inflammatory cytokines is responsible for the clinical signs of illness associated with these toxins [10,11]. Individuals who lack neutralizing antibodies to a SAg are at a higher risk of developing severe systemic disease with hypotension and organ failure, particularly if they happen to be high responders to these specific SAgs [11-13].

Four factors are thought to be required for the development of the mTSS: (1) vaginal colonization with a toxigenic strain of *S. aureus*; (2) production of TSST-1; (3) penetration of a sufficient concentration of TSST-1 across the epithelium to cause the disease; and (4) absence or insufficient titers of neutralizing antibody to the toxin. Vaginal colonization by toxigenic *S. aureus *has been reported in 1% to 4% of the populations studied [[14-18]; Parsonnet J, Tosteson A, Modern P, Wissemann K. Wissemann: Abstr. 33^rd ^Intersci. Conf. Antimicrob. Agents Chemother. abstr. 1327, 1993]. *In-vitro *studies have shown that the production of TSST-1 by toxigenic *S. aureus *is dependent on environmental factors such as the partial pressure of O_2 _and CO_2 _[19], as well as other factors such as iron concentration, pH and temperature [20-22]. Once produced, TSST-1 must then penetrate the vaginal mucosal surface. It has been shown that topical exposure to this superantigen in an ex vivo model causes an increase in mucosal permeability in a non-dose-dependent manner [23]. If TSST-1 is capable of penetrating the epithelial surfaces, it can be neutralized effectively by anti-TSST-1 antibodies [24]. Bergdoll has shown that individuals who have developed TSS tend to have lower sera antibody titers to TSST-1 than healthy individuals [8]. Studies have also shown that sera antibody titers increase with age [25] , and that the vast majority (87-100%) of the adult population has developed sera antibody to TSST-1 [[14,15,26,25]; Vergeront JM, Blouse LE, Crass, BA, Stolz SJ, Bergdoll MS, Davis JP: Abstr. Intersci. Conf. Antimicrob. Agents Chemother, abstr. 610, 1984].

Results from our previous prevalence study of 3012 healthy, menstruating women [14] supported our hypothesis that the low incidence of mTSS is a result of two factors: 1) the majority of women colonized with *S. aureus *are colonized by non-toxigenic strains, and 2) most women have positive antibody titers to TSST-1. We found 75% of *S. aureus *carriers (or 20% of the population) were colonized with non-toxigenic *S. aureus *and only 1% of the study population was colonized vaginally with toxigenic strains of *S. aureus*. Positive antibody prevalence, defined as titer ≥1:32, was high in the general study population (85%), and even higher among subjects colonized by toxigenic *S. aureus *in the nose (98%), vagina (97%), or anus (100%). Only eight percent (8%) of the study population were classified as antibody "negative," exhibiting antibody titer ≤1:4.

This follow up study was designed to determine the persistence of *S. aureus *and serum antibody among groups of menstruating women recalled from the previous study. Negative controls for *S. aureus *and serum antibody were included to determine whether subjects became *S. aureus *carriers or seroconverted during recall. Previous studies in this area have focused on the persistence of nasal carriage of *S. aureus *among different populations [27-29], as well as the classification [27], and prediction [30,31] of nasal carrier states. To our knowledge, this is the first published study to evaluate the persistence of serum antibody and *S. aureus *carriage at two additional body sites (anus and vagina).

## Methods

### Subjects

Approximately one year after completion of the previous study of 3012 subjects, published in 2005 by Parsonnet, et al., [14] subjects, a smaller subgroup was recalled and followed for an additional 6-11 months, depending upon the group assignment (Table 1). Groups consisted of both vaginal and non-vaginal carriers of toxigenic TSST-1 - producing *S. aureus*, carriers of non-toxigenic *S. aureus *and two control groups: non-carriers of *S. aureus *and subjects who were antibody negative.

**Table 1:** Description of subject groups enrolled in follow up study

Group No.	Description	Body site where *S. aureus *detected in Parsonnet 2005 **study**^**a**^	Ab present in Parsonnet 2005 **study**^**b**^	No. of subjects **enrolled**^**c**^	No. of subjects completing all visits
1	Carriers of SA TSST-1(+)	Vagina	Yes or No	14	11
2	Carriers of SA TSST-1(+)	Nose or anus	Yes	78	64
3	Carriers of SA TSST-1(-)	Any site	Yes	69	49
4	Non-carriers	None	Yes	75	58
5	Negative Ab control (Carriers or Non-carriers)	Any site	No	75	61

^a^Body sites sampled & found positive = vagina, nose and anus.

^b^Yes = anti-TSST-1 antibody titers ≥ 1:32; No = anti-TSST-1 titers ≤ 1:4.

^c ^Subjects who returned for at least one visit during follow up.

Groups 3-5 were scheduled for three equally spaced visits. Groups 1 and 2 were scheduled for four equally spaced visits because of our interest in toxigenic *S. aureus *colonization. Subjects were recalled from five geographically separate sites in North America by Hill Top Research Inc. in Cincinnati, OH; East Brunswick, NJ; St. Petersburg, FL; Scottsdale, AZ and Winnipeg, Manitoba, Canada. Recall was based on a target enrollment of 75 participants in Groups 2-5. Target enrollment for Group 1 was the total number of subjects that met the criteria (n = 33). The total number of subjects who met the Group 1 criteria (*S. aureus *TSST-1 vaginal carriers) from the previous study was very low (33 subjects/3012 total subjects). Every attempt was made to recruit all Group 1 subjects. Some of the subjects could not be located and others did not wish to participate in this follow up study.

Women were eligible for enrollment in this study if they completed the previous study and continued to meet the original enrollment criteria [14] on recall. Briefly, the criteria required subjects to be between the ages of 13-40, to be in good health, to have regular menstrual cycles (length between 21 and 35 days), to use tampons at least occasionally, and to have refrained from bathing or other activities which could alter the vaginal environment within the 2 hours prior to their scheduled visit (Table 2).

**Table 2:** Inclusion/Exclusion Criteria for the previous and this follow up study

Inclusion	Exclusion
Had a regular menstrual cycles (minimum of 21 days and maximum of 35 days);	Had participated in another clinical study
Had used tampons at least occasionally	Were pregnant, actively trying to get pregnant or suspected they were pregnant
Refrained taking a bathe or shower within the 2 hours prior to their scheduled visits	Had a gynecological abnormality as judged by the study medical personnel
Refrained from using douching substances, vaginal medications, suppositories, feminine sprays, genital wipes, or contraceptive spermacides for 48 hours prior to their scheduled visits	Had an infection of the genitals within the past 6 weeks
	Had been medically diagnosed as having diabetes, kidney failure, hepatitis, AIDS (HIV positive) or toxic shock syndrome
	Were currently taking (within the last 30 days) immunosuppressive drugs, chemotherapy, systemic antimicrobial drugs, or antifungals or antimicrobials to treat a vaginal infection

Subjects read and signed a revised informed consent document prior to the collection of any information or clinical sampling. Subjects were removed from the study if they failed to meet the inclusion criteria and satisfied any of the exclusion criteria at any time during the study.

### Study conduct

Study protocol and informed consent document were reviewed and approved by Hill Top's Institutional Review Board. Informed consent was obtained from all subjects participating in the previous prevalence study. This follow up study was conducted from February, 29, 2000 to March 29, 2001.

Procedures, methods and materials used in this study were identical to those used in the previously published study [14]. Procedures and methods are summarized in the following sections.

### Sample collection

Trained personnel at each recruitment site collected the following samples from each subject: 1) an anterior nares swab (penetration of 0.5-1.0 inch); 2) an anal swab (approximately 0.5 to 1 inch past the anal sphincter); 3) a midpoint vagina swab (approximately 3 - 4 inches into the vagina) and 4) a blood sample.

### Sample Handling

Swabs (nasal, anal and vaginal) were shipped overnight to Harvard Medical School, Channing Laboratory (Boston, MA) for isolation and phenotypic identification of *S. aureus *and TSST-1 production. Collected serum was shipped overnight to Dartmouth-Hitchcock Medical Center (Lebanon, NH) for analysis of TSST-1 antibodies. Clinical laboratories were supplied with subject number, subject's initials and area of the body sampled.

### Swab analyses

Using standard, published phenotypic methods for isolation of *S. aureus *from human samples, [14,32,33] nasal and anal swabs were streaked onto a mannitol salt agar (MSA) plate, vaginal swabs were dispersed into Amies medium with agar, streaked onto a MSA plate, incubated for 48 hrs and visualized for characteristic morphology of *S. aureus*. Colonies were enumerated, isolated onto plates containing trypticase soy agar with 5% sheep blood, and incubated for 24 hrs. Gram stain, a catalase test, and a rapid *S. aureus*-specific latex agglutination test were performed to phenotypically identify *S. aureus*.

### Determination of TSST-1 production

A previously validated and published competitive ELISA was used to quantify TSST-1 production using a membrane over agar protocol with subsequent TSST-1 quantification by ELISA [14,32,34]. After being positively identified as *S. aureus*, a heavy suspension was developed in PBS and 200 ul of the suspensions was placed on a dialysis membrane covering a 60 × 15 mm Brain Heart Infusion Petri plate. The plates were incubated in a humid chamber at 37 deg C for 24 h, washed with 1.5 mL of PBS and collected by centrifugation. Historical samples of *S. aureus *that were known not to produce TSST-1 and others that produced TSST-1 served as negative and positive controls, respectively. All *S. aureus *strains isolated at the Channing Laboratory were shipped to Dartmouth-Hitchcock Medical Center for confirmatory analyses using ELISA and/or PCR techniques [35].

### Serum analyses

An indirect antibody capture assay was used to determine the serum anti-TSST-1 IgG antibody titer of each subject. This method was used in the large seroprevalence study published by Parsonnet, et al., in 2005 [14]. Briefly, non-immune human serum from healthy volunteers and Human Immune Glogulin Intravenous IgG served as negative and positive controls, respectively. Microtiter plates coated with TSST-1 (0.5 μg/mL) and sera were diluted serially in phosphate buffered saline, incubated overnight, treated with goat anti-human IgG/alkaline phosphatase, incubated, washed, and then treated with an enzyme substrate [1 mg/mL p-nitrophenyl phosphate in 10% diethanolamine buffer]. Plates were incubated at 24°C until positive control wells reached an optical density (OD) of 1.0 (405 nm). The OD of sample solutions was determined and dilution curves were plotted for each sample. The threshold OD was determined previously by testing sera from patients with TSS caused by TSST-1. All such sera (diluted 1:4) yielded an OD of less than 0.2 times that of the positive control.

The antibody titer of each sample was calculated as the highest dilution yielding an OD greater than that of the positive control. Samples with titers of ≤ 1:4 were classified as being "negative" for antibody and those with titers of ≥ 1:32 were classified as "positive." These classifications are based on previous clinical experience with several hundred patients with either mTSS or TSST-1-induced nonmenstrual TSS. Of these patients, greater than 90% had a titer of ≤1:4 and 100% had a titer of < 1:32 (Parsonnet, unpublished observations). Titers of 1:8 and 1:16 were classified as "intermediate" (i.e., likely to contain antibodies to TSST-1, but may not be associated with protection from mTSS). Dilution curves were scrutinized by two observers, with concordance required for there to be a final antibody determination. Because the assay yielded a quantitative result (not subject to observer bias), there was no inter- or intra-observer variation in the antibody result.

### Classification of *S. aureus *carriage

Carriage was classified as transient, intermittent or persistent based on calculations of carrier index in order to compare results to others in the literature [27]. Subjects were included if they had completed a minimum of three post initial visits. The carrier index (CI) was calculated as the number of swabs with positive culture of *S. aureus *relative to the total number of swabs taken × 100. Subjects with carrier indices of 0 - 24% were classified as transient, between 25 - 74% were classified as intermittent, and 75 - 100% were considered persistent carriers. This classification was an arbitrary designation and was use to simply explain the data.

## Results

### Demographics of subjects

The geographical distribution of the 311 subjects enrolled in the study was similar to that for the previous study [14], except that the percentage of participants from Arizona, Florida and Manitoba was slightly higher. The distribution was: Arizona, 24% (*n *= 76); Florida, 22% (*n *= 68); New Jersey 13% (*n *= 41); and Ohio, 16% (*n *= 50) (United States) and Manitoba, 24% (*n *= 76) (Canada). The mean age of subjects enrolled in each group varied between 26.8 and 29.0 years of age. The ethnic/racial distribution varied between the five groups as follows: Caucasian: 77 - 90%; Black: 4 - 15%; Hispanic: 0 - 5%; Asian: 0 - 4%.

### Persistence of *S. aureus *carriage

Results in Table 3 show that nasal carriage of *S. aureus *is more persistent (35.5% and 41.0% groups 2 and 3) than vaginal or anal carriage, regardless of the strain. Vaginal and anal carriage of *S. aureus *is more transient (ranging from 55 to 77% of group) than nasal carriage. Seventeen subjects assigned to Group 4 (the negative control for *S. aureus*) were found to be culture-positive for non-toxigenic *S. aureus *and four of these showed persistent carriage.

**Table 3:** Classification of *S. aureus *colonization types in subjects completing a minimum of three subsequent visits

Group No.	Sample location	*S. aureus*	No. of subjects	% Transient carriers (#subjects) **CI**^**a **^**= 0 -24%**	% Intermittent carriers (#subjects) **CI**^**a **^**= 25 -74%**	% Persistent carriers (#subjects) **CI **^**a **^**= 75-100%**
1	Vagina	Toxigenic	13	76.9% (10)	23.1% (3)	0% (0)
2	Nasal	Toxigenic	61	29.5% (18)	29.5% (18)	41.0% (25)
2	Anal	Toxigenic	12	66.7% (8)	33.3% (4)	0% (0)
3	Vagina	Non-toxigenic	22	54.5% (12)	40.9% (9)	4.5% (1)
3	Nasal	Non-toxigenic	31	29.0% (9)	35.5% (11)	35.5% (11)
3	Anal	Non-toxigenic	14	64.3% (9)	28.6% (4)	7.1% (1)
4	Any site	Non-toxigenic	58	70.7% (41)	22.4% (13)	6.9% (4)
4	Any site	Toxigenic	58	94.8% (55)	5.2% (3)	0% (0)

^a^CI = carrier index, the number of swabs with positive culture of *S. aureus *relative to the total number of swabs taken × 100

### Persistence of serum antibody to TSST-1

**Figure 1:**
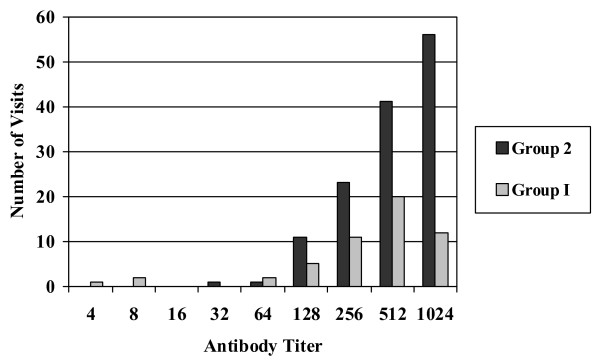
**Anti-TSST-1 antibody titers for Groups 1 & 2**. A histogram of antibody titers expressed as a two-fold serial dilution starting at 1:4 to ≥ 1024 versus total number of subject visits.

**Figure 2:**
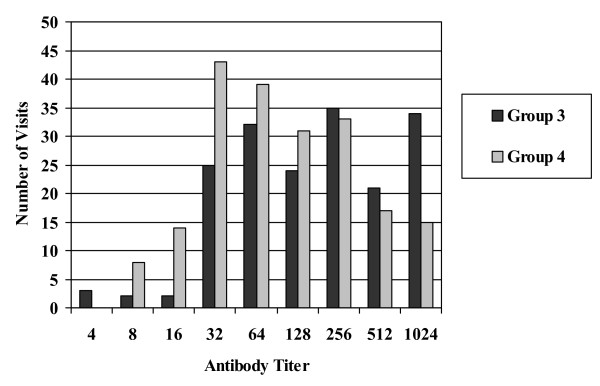
**Anti-TSST-1 antibody titers for Groups 3 & 4**. A histogram of antibody titers expressed as a two-fold serial dilution starting at 1:4 to ≥ 1024 versus total number of subject visits.

Serum antibody titers distributions from Group 1 and 2 (Figure 1) were found to be skewed toward higher titers during the follow up study, with the vast majority of visits showing titers ≥1:64. Carriers of non-toxigenic *S. aureus *(Group 3) and non-carriers (Group 4) (Figure 2) continued to show a broad distribution of antibody titers during the follow up study with antibody titers ≤ 1:64 at a significant number of visits. In total, twenty-one subjects in Groups 3, 4, and 5 became colonized with TSST-1 producing *S. aureus *during the course of the follow up study either by converting from non-toxigenic *S. aureus *or by acquiring a toxigenic *S. aureus *colony at one of the body sites. None of these subjects showed symptoms of toxic shock syndrome. In addition, these subjects were generally found to have sera anti-TSST-1 antibody distributions similar to carriers in Groups 1 and 2.

Five subjects from Group 5 seroconverted during the course of this study. Four subjects seroconverted between the end of the previous study [14] and the start of this study (approximately 1 year). One subject seroconverted between visit #3 and visit #4 of the follow up study (Table 4). Their ages ranged from 15 to 36, and none experienced TSS-like symptoms. No evidence of TSST-1 producing *S. aureus *was found in the body sites cultured from the four subjects, but it was detectable in the subject who converted between visit #3 and visit #4. This corresponded to the time interval in which serum anti-TSST-1 antibody titer increased from ≤1:4 to 1:128.

**Table 4:** Antibody titer by visit for subjects in Group 5 that seroconverted

Age of subject	**Ab**^**a **^**titer previous study**	**Ab**^**a **^**titer during follow up study**		
		
	Visit #1	Visit #2	Visit #3	Visit #4
15	≤1:4	1:512	1:512	1:1024
18	≤1:4	1:128	1:64	1:64
29	≤1:4	1:64	1:64	N.C.^b^
26	≤1:4	1:128	1:128	1:512
36	≤1:4	≤1:4	≤1:4	1:128^c^

^a^Anti-TSST-1 antibody.

^b^Not collected; subject did not return for final visit.

^c^TSST-1 producing *S. aureus *isolated from sample taken at this visit.

## Discussion

Results of this study among healthy, menstruating women indicate that the distribution of carriage types vary more by body site than by the type of *S. aureus *strain (toxigenic or non-toxigenic). Nasal carriage of *S. aureus *appeared to be persistent (41% and 35.5%), whereas vaginal and anal carriage appeared to be more transient. Compared to findings from other studies summarized by VandenBergh [27], nasal carriage was more persistent in this study, which may be due to the fact that the subjects in these groups were selected based on their initial *S. aureus *carriage. A recent article by Nilsson and Ripa [36] determined the frequency and persistence of *S. aureus *colonization in the nose and throat in hospital patents and staff. These results demonstrated a nasal carriage rate of 31% and 36%, respectively and a more frequent *S. aureus *throat colonization of 46% and 54%. This may indicate the throat to be the primary source for subsequent *S. aureus *at other anatomical sites.

The higher persistence of *S. aureus *nasal carriage may reflect higher colonization of *S. aureus *in the nasal environment than the other body sites. Early experiments suggested that *S. aureus *binding to mucin may be critical for colonization of the nasophyngeal mucosa [37]. It has been suggested that the presence of type I cytokeratin 10 in the nasal epithelium allows clumping factor B (clfb) associated with *S. aureus *to bind to the epithelium and promote nasal colonization [38]. Another contributing factor to colonization frequency may be associated with human gene polymorphisms. An article published in 2006 by van den Akker [39] showed a clear association between persistent *S. aureus *nasal colonization and the glucocorticoid (GC) gene receptor. Carriers of the GC polymorphism were shown to increase the risk of persistent *S. aureus *nasal carriage by 80%.

The absence of persistent carriage in the vagina may reflect low colonization due to the lack of type I cytokeratin 10 in the mucosal epithelium or to changes in the vaginal environment during the menstrual cycle. Previous studies of vaginal biopsies using monoclonal antibodies showed the lack of cytokeratin 10 on the vaginal epithelium [C. Davis, A Kanti, M. Hansmann, J. Flood, C. Squier and J. Krueger, Abstr. Intersci. Conf. Antimicrob. Agents Chemother. abstr. B-1111, 2002]. Changes in the vaginal environment related to the menstrual cycle, such as levels of iron, O_2_, CO_2_, pH, hormones [19-22], could also affect the total number of bacteria present or the number of colonizing species.

We speculate that the absence of persistent carriage in the anus may be related to lack of cytokeratin 10 binding sites (a marker associated with keratinizing cells) in the columnar epithelium, presence of intestinal mucus, and/or competition from the large population of microorganisms (including lactic acid bacteria) in the human intestine [40,41].

The distribution of serum antibody titer among Group 1 subjects (Figure 1) was found to remain skewed toward higher titers during subsequent visits even though the number of positive culture isolations of toxigenic *S. aureus *dropped significantly during this part of the follow up study. This was also found to be true among Group 2 subjects (Figure 1); however, Group 2 also had a higher level of reisolation of toxigenic *S. aureus *during the subsequent visits. This seems to suggest that colonization at some point in time is sufficient to elevate antibody titers and those levels appear to be persistent. Antibody titer distributions among Groups 3 and 4 (Figure 2) showed trends similar to the original 3012 subjects' antibody titer distribution.

Five subjects with no initial serum antibody became seropositve (titer ≥ 1:32) over the course of the two and a half year study for a seroconversion rate of 7% of subjects. It is important to note that two of the five subjects were 18 years of age or younger. In addition, none of these subjects experienced TSS-like symptoms. These findings suggest that women can become seropositive at any stage of life and the serum antibody can develop as a result of environmental exposures that do not result in TSS-like symptoms.

## Conclusions

Nasal carriage of *S. aureus *appeared to be persistent and the best predicator of subsequent colonization, whereas vaginal and anal carriage appeared to be more transient. It is interesting that at least 29.3% (17/58) of previous stated as non carriers showed to be positive during the follow up visits. This reinforces the concept that the carrier state for the human is not always absolute and this has been noted by other investigators [42]. A limitation of our study is that swab samples were analyzed in singlet, so that inter-observer variation in detection of *S. aureus *could not be determined; conventional methods for bacterial isolation and identification were employed, however, for which reason we are confident in the quality of our data.

Serum IgG levels against TSST-1 has also been found to be higher in persistent carriers than in non-carriers of *S. aureus*[42]. From these findings, it appears that antibody titers in women found to be colonized with toxigenic *S. aureus *remained skewed toward higher titers whether or not the colonies were found to be persistent or transient in nature. This suggests that colonization at some point in time is sufficient to elevate antibody titer levels and those levels appear to be persistent. Results also indicate that women can become seropositive (7% seroconversion rate) without experiencing signs or symptoms of toxic shock syndrome.

Colonization of *S. aureus *TSST-1 and the absence of antibodies to TSST-1 are important in the pathogenesis of mTSS. Other factors not studied in the report include specific V beta 2 TCR (T cell receptor) expansion and human leukocyte antigen (HLA) haplotype [43,44].

## Competing interests

JP: Received a grant for this research work.

MH: Is an employee of the Procter & Gamble Co and holds shares of stock in Procter & Gamble.

JLS: Is a retired employee of the Procter & Gamble Co and holds shares of stock in Procter & Gamble.

MLD: Received a grant for this research work.

AMD: Received a grant for this research work.

PAM: Received a grant for this research work.

MBJ: Is a retired employee of the Procter & Gamble Co and holds shares of stock in Procter & Gamble.

JEW: Hilltop Research received financial support for clinical work. JEW was employed as the clinical investigator and executed the clinical portion of the study.

ABO: Received a grant for this research work.

## Authors' contributions

JP was co-investigator and provided data interpretation. AO was co-investigator and provided data interpretation. JW was a co-investigator and provided clinical support of the trial. MH provided study design & clinical support. JS provided study design & clinical support. MD provided microbiological support. AD provided microbiological support. PM provided antibody titer analyses. MJ provided statistical analyses. All authors read and approved the final manuscript.

## Pre-publication history

The pre-publication history for this paper can be accessed here:

http://www.biomedcentral.com/1471-2334/10/249/prepub
